# 89 Association of brucellosis and ankylosing spondylitis: a pediatric case report

**DOI:** 10.1093/rheumatology/keac496.085

**Published:** 2022-10-07

**Authors:** Djohra Hadef, Oum Keltoum Mansouri, Hayat Behamma, Nourelhouda Benbouzid, Imane Boukhalfa, Hind Bougherara, Hadda Rouichi, Khaoula Gourmat, Khadidja Bezziou, Kamel Serhane

**Affiliations:** 1 Department of Pediatrics, CHU Batna, Algeria; 2 Faculty of Medicine, University of Batna 2, Algeria

## Abstract

**Introduction:**

Ankylosing spondylitis is a common chronic rheumatic disease in male young adults. Brucellosis is a worldwide zoonosis and remains endemic in several Mediterranean countries, which may have features similar to other diseases, leading to serious problems of differential diagnosis. This case report described a case of a 14-year-old male diagnosed with ankylosing spondylitis whose exploration reveals associated subacute brucellosis.

**Methods and results:**

A 14- years- old boy from Batna, born of a first degree consanguineous marriage, and the 2nd of a sibling of 05 children. He had no relevant past medical history. He complained of chronic polyarthritis of three years, affecting the large joints: hip, knees, ankles, elbows and wrists as well as the cervico-dorsolumbar spine. Pain on palpation of the tendon insertion zones without fever. Delayed growth in weight and height was found. He was misdiagnosed at disease onset as Rheumatic fever treated by Extencillin with vitamin D3 supplementation but no improvement was noticed.

The Ultrasound examination of the joints revealed moderate effusion with synovial thickening of the hip (hip synovitis). CT scan of the cervical-dorsal-lumbosacral spine revealed a bilateral erosive sacroiliitis with cervical ankylosis of the posterior apophyseal joints at C2-C3, C3-C4, and C4-C5 levels. A biological inflammatory syndrome was found. Brucellosis serology (Rose Bengal and Wright) was positive twice (IgM and IgG). Quantiferon test was negative.

The treatment of the Brucellosis was started (Doxicyclines, Gentamycin, and Rifampicin). Non-steroidal anti-inflammatory drugs were also prescribed.

**Conclusion:**

Brucellosis is a differential diagnosis of spondyloarthritis. However, an association of both diseases might exist and should be adequately managed.

**Disclosure of Interest:**

None declared

